# An Improved Method for TAL Effectors DNA-Binding Sites Prediction Reveals Functional Convergence in TAL Repertoires of *Xanthomonas oryzae* Strains

**DOI:** 10.1371/journal.pone.0068464

**Published:** 2013-07-15

**Authors:** Alvaro L. Pérez-Quintero, Luis M. Rodriguez-R, Alexis Dereeper, Camilo López, Ralf Koebnik, Boris Szurek, Sebastien Cunnac

**Affiliations:** 1 UMR 186 Résistance des Plantes aux Bioagresseurs, Institut de Recherche pour le Développement, Montpellier, France; 2 Biology Department, Universidad Nacional de Colombia, Bogotá D.C., Colombia; University of the West of England, United Kingdom

## Abstract

Transcription Activators-Like Effectors (TALEs) belong to a family of virulence proteins from the *Xanthomonas* genus of bacterial plant pathogens that are translocated into the plant cell. In the nucleus, TALEs act as transcription factors inducing the expression of susceptibility genes. A code for TALE-DNA binding specificity and high-resolution three-dimensional structures of TALE-DNA complexes were recently reported. Accurate prediction of TAL Effector Binding Elements (EBEs) is essential to elucidate the biological functions of the many sequenced TALEs as well as for robust design of artificial TALE DNA-binding domains in biotechnological applications. In this work a program with improved EBE prediction performances was developed using an updated specificity matrix and a position weight correction function to account for the matching pattern observed in a validation set of TALE-DNA interactions. To gain a systems perspective on the large TALE repertoires from *X. oryzae* strains, this program was used to predict rice gene targets for 99 sequenced family members. Integrating predictions and available expression data in a TALE-gene network revealed multiple candidate transcriptional targets for many TALEs as well as several possible instances of functional convergence among TALEs.

## Introduction

Transcription activator-like effectors (TALEs) belong to a family of bacterial proteins initially identified in the *Xanthomonas* genus of plant pathogens. TALEs are translocated into plant cells via the bacterial type III secretion system (T3SS). They operate by mimicking the activity of plant transcription factors and bind to promoter regions of host plant genes. When TALEs contribute to virulence, the induction of their cognate target gene, referred to as a susceptibility gene, enhances the fitness of the bacteria in plant tissues [1]–[3]. Occasionally, TALEs also specify incompatibility (resistance) on certain plant host genotypes. This occurs by two described mechanisms: either a TALE induces the expression of a plant resistance gene, also termed executor gene (dominant resistance), or a DNA polymorphism in the upstream region of a TALE susceptibility gene prevents target site recognition (recessive resistance) [1].

TALEs have highly conserved amino acid sequences containing T3SS secretion and translocation signals in their N-terminal region, a central DNA-binding domain, nuclear localization signals and a transcriptional activation domain in their C-terminal region [1], [2]. TALEs differ mostly in their central region consisting of a number (10–25) of tandemly arranged repeats of typically 34 amino acids each with hypervariable di-amino acids at positions 12 and 13, called RVDs (Repeat Variable Diresidues) [4], [5]. The binding of TALEs to DNA sequences is highly specific due to unique combinations of RVDs. Individual RVDs selectively associate with individual nucleotides depending on the nature of the amino acids in the diresidue. For example, a TALE repeat with the RVD “HD” (His and Asp at positions 12 and 13) binds preferentially to cytosine [4], [5]. Moreover, the sequence of RVDs determines the preferred DNA sequence for binding in a co-linear fashion. The elucidation of this TALE-DNA specificity “code” enabled the first predictions of TALEs binding sites in plant genomes [4], [5]. More recently, the crystal structures of TALEs showed that their DNA-binding domain wraps around the DNA double helix helicoidally with the RVDs in the innermost part of the complex in close proximity with their cognate nucleotide on the DNA molecule [6]–[9]. It was also shown that the two amino acids in the RVD have different roles in the interactions. The first amino acid in the RVD (the 12th in each repeat) does not directly contact DNA but stabilizes the local conformation of the RVD loop, while the second amino acid determines specificity by interacting directly with the nucleotide via hydrogen bonds or van der Waals interactions [7], [9].

The discovery of the TALE-DNA code was a milestone not only in plant-pathology but also in biotechnology. By using the binding specificities of the best characterized RVDs (HD−> C, NI−> A, NG−> T, NN−> G, NS−> N) and taking advantage of the modular nature of TALEs, researchers have been able to engineer artificial TALE proteins with any desired specificity to induce the expression of individual genes, not only in plants but also in other eukaryotes [10]. Furthermore, the repeat regions of TALEs have been fused with other protein domains of interest, notably nucleases, thus allowing the specific editing of desired genomic regions [10]. Custom TALE domains coupled with repressor domains for specific transcriptional control have also been engineered [11]. Some of these constructions are already being used to produce improved phenotypes (*e.g*, disease resistance) in plants and animals, and the potential applications of this technology are remarkable [12]–[14].

The *Xanthomonas* genus comprises 27 species that collectively cause major diseases on more than 400 plant hosts, including a wide variety of economically important crops, such as rice, citrus, banana, cassava, cabbage, tomato, pepper and beans [15]. There are currently 104 *Xanthomonas* genome sequences available from 11 species (http://www.xanthomonas.org/) and this number is likely to increase dramatically in the near future. *Xanthomonas* strains harbor quantitatively and qualitatively different repertoires of TALEs, and the total number of sequenced TALEs available in databases reaches roughly 100 (http://www.xanthomonas.org/). Despite great advances in TALE-based biotechnology, the collective role of TALEs during infection has not been systematically investigated and only a few transcriptional targets have been validated in host plants [4], [5], [16]–[21]. Yet, the ability to predict binding sites for TALEs is key to a better understanding of the disease process and to develop genetic resistance strategies which are greatly needed considering the impact of *Xanthomonas* on agriculture worldwide [22].

Currently, a single TALE target prediction algorithm based on the published TALE-DNA code [5] has been released and is available in the TALE-NT suite [23]. Despite its ability to correctly identify previously unknown targets of some TALEs [5], it fails to detect the genuine targets of others (see Results section). This is particularly true for several TALE-target pairs that exhibit RVD-nucleotide association patterns deviating from expected matches based on the documented specificities [5], [16]. The effects of such imperfect RVD-nucleotide pairings on TALE activity still remain elusive and therefore represent an obstacle for the accurate prediction of TALE Effector Binding Elements (EBEs).

In this work, we used the available literature on TALE-RVD specificities from experimentally validated TALE-DNA interactions to develop new programs for TALE binding sites prediction. Our software outperformed the TALE-target finder module of the TALE-NT suite in differentiating positive and negative TALE-DNA interactions. It was subsequently employed to predict rice microarray data-supported new binding sites for TALEs from various *X. oryzae* strains. Because we had access to TALE repertoires of both the xylem vessel-associated *X. oryzae* pv. *oryzae* (*Xoo*), the causative agent of the bacterial leaf blight, and the mesophyll-associated *X. oryzae* pv. *oryzicola* (*Xoc*), the causative agent of the bacterial leaf streak, we could also compare the plant candidate target sets of these pathovars with contrasting tissue specificity. The resulting predictions reveal a putative network for TALE-gene interactions with several examples of functional convergence within and across strains.

## Results

### New Approaches for EBE Predictions that use a Modified RVD-nucleotide Association Matrix

In this work, two programs named Talvez and Storyteller were designed to predict TAL effectors binding sites in plant genomes. Similar to the reference target finder program from the TALE-NT suite [23], hereon referred to as Talent, both programs use a RVD-nucleotide association matrix to convert a sequence of RVDs for a given TALE into a positional weight matrix (PWM). However, we first refined the previous RVD-nucleotide association model published in [4], [5] to account for more recent insight into RVD-nucleotide specificities [7], [9]. In our updated matrix (Table S1), identical counts were assigned to all RVDs sharing the same second hyper variable residue (e.g. HG, NG), since it has been shown that this residue only is responsible for nucleotide specificity [7], [9]. In addition, considering that cytosine binding at position 0 is possible and functional, albeit with lower activity [9], [18], prediction of cytosine binding at this position was allowed but was assigned a lower value than thymine. For predictions, both Talvez and Storyteller first convert a sequence of RVDs into a PWM based on this updated RVD-nucleotide association matrix. Talvez then uses the PWM to scan and score all possible EBEs with a log-likelihood function developed originally to study plant transcription factors binding sites [24]. In contrast, Storyteller uses the PWM to generate a set of possible EBEs and constructs a hidden Markov model that is then fed to the program HMMER [25] to scan DNA sequences. (*see Materials and Methods, program descriptions*).

### Comparative Analysis of the Performances of the EBE Predictors in a Curated Set of Experimentally Validated TALE-DNA Interactions

**Figure 1:**
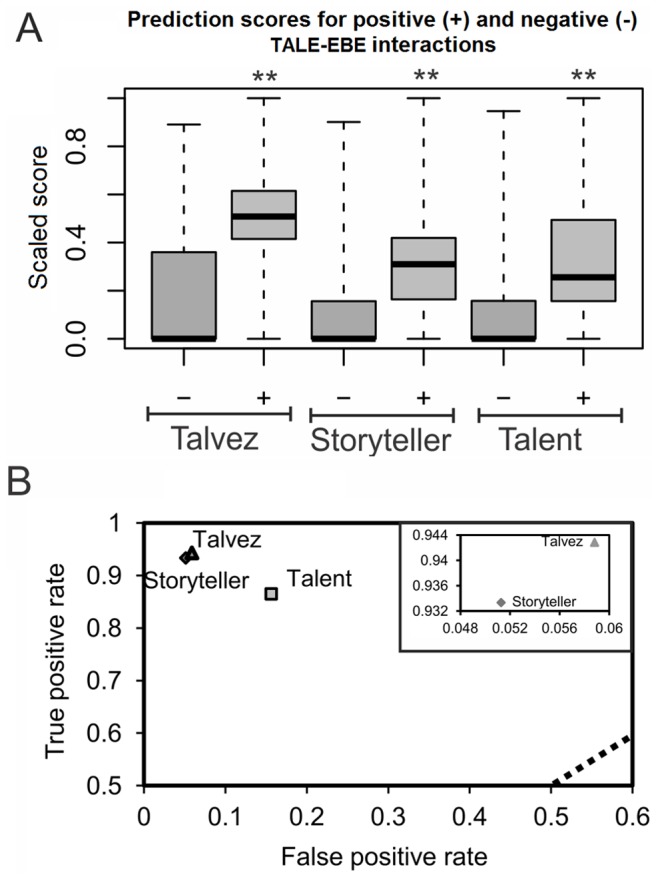
Performances of the EBE prediction software in the TALE-EBE validation set. (**A**) Boxplot showing the median (thick line), the lower and upper quartiles (box) and the minimum and maximum (whiskers) of the prediction scores for the set of positive (+) and negative (−) control TALE-DNA interactions using three programs for EBE prediction. Scores were scaled down according to the maximum score on the set to facilitate comparison. Talent scores were scaled x^−1^ since they follow an inverse scale relative to the other programs, this transformation maintains data structure. ** Indicates significant positive vs. negative differences (one-tailed t-test p-value<0.001). (**B**) ROC graph showing the true positive and false positive rate of the three EBE predictors based on validation set screenings. Dashed line indicates the theoretical performance of a random classifying program where true positive rate = false positive rate. The inset in the upper right corner shows the rates for Talvez and Storyteller at a higher scale to highlight the differences between the two programs.

To compare the performances of the EBE prediction programs, a validation set was first established (Table S2). It is composed of 72 experimentally confirmed control TALE-EBE interactions obtained from the literature for a total of 22 TALEs [4], [5], [14], [16]–[21], [26]–[30]. Of these, 35 were classified as positive interactions because the considered EBE has been shown to mediate specific transcriptional induction of downstream sequences in the presence of its cognate TALE (e.g., by beta-glucuronidase [GUS] reporter assays). Another 37 interactions were classified as negative. These involved mutated EBEs that in contrast to the wild type sequences were demonstrated to be non functional in the same experiments. Next, the validation set was screened using Talvez, Storyteller, and Talent as a reference. All three programs were able to score positive interactions significantly higher than negative ones (one-tailed t-test, p-value<0.001) (Figure 1A). Accordingly, in the ROC graph of Figure 1B, the three programs localize well above the random guess diagonal and close to the upper left corner, which denotes high rates of true positives (up to 0.942% for Talvez) and low rates of false positives (as low as 0.051% for Storyteller). Both Talvez and Storyteller appear better than Talent for both performance metrices and display comparable rates even though Storyteller is slightly more conservative than Talvez on this validation set (inset of Figure 1B).

### Comparative Analysis of EBE Predictors’ Ability to Rank Positive Control Target Genes in Complete Sets of Promoters from Plant Genomes

Next we sought to comparatively evaluate the three predictors in a realistic situation recapitulating a typical application of the software. To this end, the predictors were tested for their ability to detect and assign a high score to genuine TAL targets in complete sets of promoter sequences, which exhibit a very different prediction class than our validation set. We first selected those TALEs from the validation set (AvrXa27, AvrXa7, PthXo1, PthXo3, PthXo6, PthXo7, Tal1C, Tal9a and TalC from *X. oryzae* strains; Hax2 from *X. campestris* pv. *armoraciae*) that have at least one genuine gene target and associated EBE in the genome of the host plants *Oryza sativa* cv. Nipponbare or *Arabidopsis thaliana* Col0 (Table 1 and Table S2). This subset of TALEs served to screen the promoters (500 bases before the translation start site) of all annotated protein-coding loci of the appropriate plant genome. Table 1 details for each of these TALEs the ranking of the score of its cognate positive control target gene(s) among all predictions for this particular TALE, broken down by prediction program. For five out of eleven predictions (AvrXa27, AvrXa7-LOC_Os11g31190, Hax2, PthXo3, PthXo6), Talvez ranked the control target better than the other programs. For five of them (AvrXa7-LOC_Os04g19960, PthXo1, Tal1c, Tal9A, TaLC), it performed as well as the other program(s). For one prediction (PthXo7- *OsTFIIAγ1*), Talent performed the best (Table 1). Intriguingly, five of the control TALE-EBE pairings, namely, AvrXa27-*Xa27*, AvrXa7-*Os11N3*, AvrXa7-LOC_Os04g19960, PthXo3-*Os11N3* and Hax2-*PAP1* could not be detected or their scores ranked extremely low (Table 1). It has been noted before that in these interactions, the EBE sequence deviates from the canonical RVD-nucleotide association model [4], [5], [16], [31] but it is not currently well understood how these pairings function nonetheless.

**Table 1:** Comparison of EBE predictors rankings of known TALE targets in genomic searches.

TALE	Strain	Target Gene Name	Locus ID	EBEdistance	Reference	Talvezrank	Storyteller rank	Talent rank
**AvrXa27**	Xoo PXO99^A^	*Xa27*	LOC_Os06g39810	−84	[4], [21]	674	Ø	3186
**AvrXa7**	Xoo PXO86	*Os11N3*	LOC_Os11g31190	−259	[16], [26]	261	344	443
**AvrXa7**	Xoo PXO86		LOC_Os04g19960	−60	[14]	Ø	Ø	Ø
**Hax2**	Xca 5	*PAP1*	AT1G56650	−130	[4]	231	438	Ø
**PthXo1**	Xoo PXO99^A^	*Os8N3*	LOC_Os08g42350	−251	[4], [17], [26]	1	1	1
**PthXo3**	Xoo PXO61	*Os11N3*	LOC_Os11g31190	−261	[16]	752	Ø	Ø
**PthXo6**	Xoo PXO99^A^	*OsTFX1*	LOC_Os09g29820	−136	[4], [19]	1	2	2
**PthXo7**	Xoo PXO99^A^	OsTFIIAγ1	LOC_Os01g73890	−469	[4], [19]	9	6	2
**Tal1c**	Xoo PXO99^A^	*OsHen1*	LOC_Os07g06970	−217	[5], [29]	1	1	1
**Tal9A**	Xoc BLS256	*OsHen1*	LOC_Os07g06970	−206	[5], [29]	1	1	1
**TalC**	Xoo BAI3	*Os11N3*	LOC_Os11g31190	−319	[18]	1	2	1

The table reports the ranking of the EBE corresponding to a control target gene in the set of predicted EBEs produced after searching *Arabidopsis* or rice promoters with a control TALE query and one of the three prediction program. The background shading of the rankings is coded as follow: white, best ranking among the predictors; light grey, detected but ranks below the best predictor; dark grey and Ø denote that no EBE was detected above cut-off score. EBE location refers to the distance between the first base (5′) of the EBE and the annotated translation start of the gene. The references of the literature supporting each TALE-control gene target pair are indicated in brackets.

From our comparative analysis of the relative performances of the three algorithms, we conclude that both Storyteller and Talvez, which rely on the same novel RVD-nucleotide association matrix, outperformed the reference predictor Talent in terms of true and false positive rates when evaluated in the framework of our curated validation set. Furthermore, in searches against the entire rice and *Arabidopsis* promoter sets for positive control TALE-gene target pairs, Talvez exhibited a higher detection sensitivity and better rankings than the two other programs. Consequently, the Talvez predictor was selected for subsequent work on refining the prediction approach.

### Implementation of a Position Correction Parameter to Tolerate RVD-nucleotide Mismatches Towards the C Terminal End of RVD Sequences

**Figure 2:**
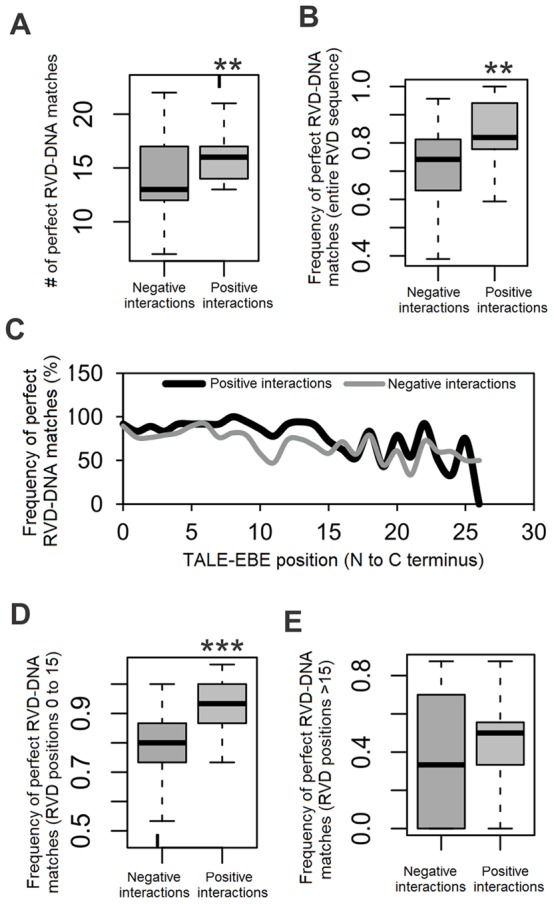
Distribution of perfect matches (PM) in the TALE-EBE validation set. (**A**) Box plot of the distribution of the number of perfect RVD-nucleotide matches computed for individual negative and positive control TALE-EBE pairs. (**B**) Distribution of perfect match frequency of individual control TALE-EBE pairs. The frequency corresponds to the ratio of the number of perfect RVD-nucleotide matches to TALE length expressed in number of RVD. (**C**) Frequency of perfect matches across TALE-DNA positions. The frequency corresponds to the ratio of the number of perfect RVD-nucleotide at the considered position to the total number of RVD-nucleotide pairs at this position in TALE-EBE pairs of the positive or negative control set. (**D**) Frequency of perfect RVD-nucleotide match between positions 1 and 15 ( = number of PM/15). (**E**) Frequency of perfect match for TALE-DNA positions beyond 15 ( = number of PM/(length-15)). The p-value of the corresponding two-tailed Wilcoxon test in this comparison is 0.371. ** significant differences, one-tailed Wilcoxon test p-value<0.001; *** significant differences one-tailed Wilcoxon p-value<1e-7.

In order to further improve predictions, we asked why, as highlighted above, some genuine EBE had low ranks or were not detected at all in our initial analysis. For this, we systematically inspected the quality of RVD-nucleotide matches for each TALE-EBE pairing in our validation set of 74 interactions. We defined as perfect matches (PM) those instances of RVD-nucleotide matches that conform to the most probable pairing specified in the updated RVD-nucleotide association matrix (cells with a black background in Table S1) as opposed to other possibilities. Not unexpectedly, we found that positive interactions had significantly more total PM than negative interactions (Figure 2A). The same held true when normalizing for the number of RVDs in each TALE (Figure 2B). We subsequently examined the PM distribution along the aligned RVD/nucleotide sequences by computing the PM frequency for each position in the TALE-EBE interactions (numbered from N- to C- terminus in the TALE repetitions, and 5′ to 3′ in the EBE in Figure 2C). As shown in Figure 2D, we observed that the PM frequency in the first 15 positions was statistically different between negative and positive interactions. In contrast, there was no difference in PM frequency when considering the region after position 15 in the 15 out of 21 TALEs of our validation set that had more than 15 RVDs. These observations suggested that the RVD-nucleotide match pattern in the distal part of the RVD sequence-EBE alignment holds little useful information for distinguishing productive TALE-DNA interactions. We reasoned that it may be counterproductive to strongly penalize suboptimal RVD-nucleotide pairings in distal regions of the alignment as this may unnecessarily diminish the overall score of legitimate hits relative to background. We therefore added a “position correction” parameter to the Talvez program in order to buffer this potential effect and to ultimately improve the performances of our prediction model. The value of the position correction parameter corresponds to a RVD position after which a scaled down matrix for RVD-DNA specificities is used instead of the standard matrix. In our scaled down matrix, non-perfect matches are no longer penalized and are assigned a count value slightly lower than perfect matches (See the Material and methods section and Table S3).

### The Position Correction Parameter Improves EBEs Prediction

**Figure 3:**
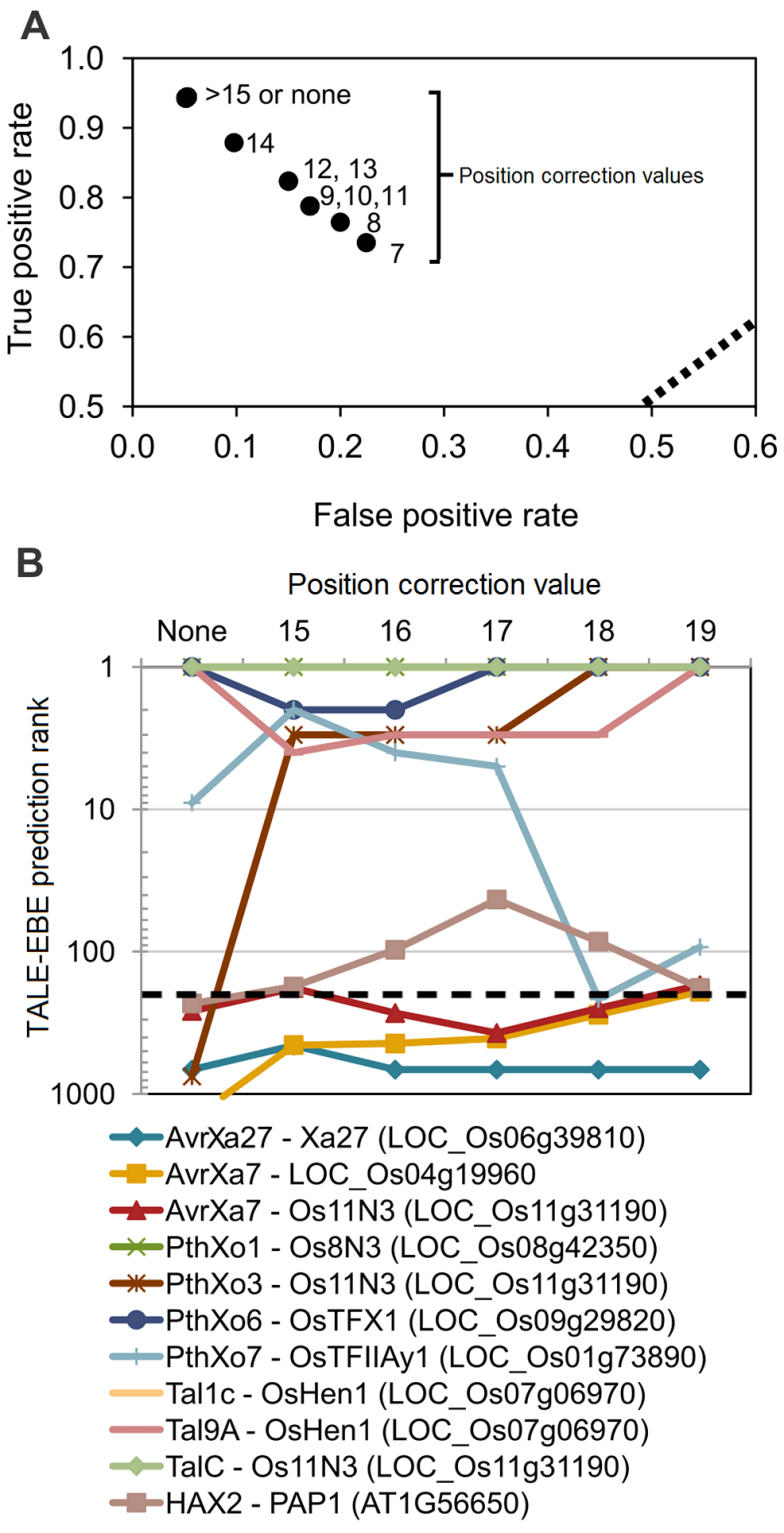
Effects of the Talvez position correction parameter on performances. (**A**) ROC graph showing true positive and false positive rates obtained by screening the validation set with a range (7–25) of position correction values. The data points for positions above 14th as well as for Talvez without position correction (labeled with “None”) all superpose on the left uppermost point. (**B**) Rankings of positive control TALE targets among genes predicted to contain EBEs in their promoter regions after screening the *Arabidopsis* and rice genome with Talvez and position correction parameter value varying between 15 and 19 as well as without position correction. The color coding of the various TALE-target pairs is described in the legend beneath the plot. Rankings for positions above 19 were similar to those without position corrections and were omitted here. The dashed line corresponds to rank values equal to 200.

Next, we addressed the effect of the position correction feature on both false positive and false negative rates on our validation set (Table S2) by varying the position correction parameter of Talvez between 7 and 26. In Figure 3A, position values below 15 produced poorer predictions than without position correction. This probably reflects the loss of information due to a premature usage of the less discriminatory scaled down RVD-nucleotide specificity matrix. In contrast, for positions above or equal to 15, the rates of true positives and false positives were robust and remained similar to the values obtained without correction (Figure 3A), indicating that in this context, position correction had no measurable effect on the program’s performance. Furthermore, as shown in Figure 3B, the complete sets of rice and *Arabidopsis* promoters were screened as before to assess the ranking of control genomic targets upon position correction. Overall, a position correction value of 19 substantially improved the ranking of positive control targets relative to no correction at all. Moreover, 6 of these 11 targets were ranked first among the predicted target promoters (Figure 3B). Notably, with our dataset, this position correction value appeared to be a good compromise in the sense that it was the only one that, with the exception of *Xa27*, ranked all positive control targets within the top 200 best predictions of their cognate TALE (Figure 3B).

In conclusion, these results suggested that optimal RVD-nucleotide pairings in the C-terminal region of TALEs DNA-binding repeat domain may contribute to a lesser extent than the N-terminal region to productive TALE-DNA associations. This property was integrated into Talvez by not penalizing mismatches after a certain RVD. Benchmarking of the position correction value in our validation and genomic control sets revealed that values above position 15, and particularly position 19 provided an appreciable gain in prediction performances over no position correction; hence it was subsequently employed when searching for TALE targets in the rice genome with Talvez.

### Defining the Search Parameters for the Comprehensive Identification of High Quality Candidate Targets of *X. oryzae* TALEs in Rice

TALEs are central to the interaction between many *Xanthomonas* strains and their respective host plant. They are often the primary molecular factor explaining the outcomes of both compatible (disease) and incompatible (resistance) interactions [32]. The large number of TALE genes found in the genome of some *X. oryzae* pathovars (e.g. 17 for *Xoo* PXO99^A^ and up to 32 for *Xoc* BLS256) [33] makes them atypical relative to other species of the genus and raises the question of the biological significance of such enlarged genomic TALE repertoires. The availability of complete TALE sets in the genome of *X. oryzae* strains as well as numerous TALE sequences from a variety of other strains prompted us to use our prediction algorithm in order to comprehensively investigate the sets of high quality candidate TALE targets in the *Oryza sativa* cv. Nipponbare genome. To this end, we cross checked Talvez predictions with experimental evidence of candidate target gene induction during compatible interactions in publicly available microarray data as a good estimate of true direct up-regulation of a gene by a TALE in a rice susceptible background.

Talvez was used to identify candidate target genes for all sequenced TALEs from Asian *Xoo* and *Xoc* strains with associated microarray data of rice gene responses to infection in databases (*Xoo* PXO86, *Xoo* PXO99^A^, *Xoo* MAFF311018, *Xoc* BLS256, 69 TALEs were used and 20 microarray comparisons; see Table S4 and Table S5). To minimize the false positive rate, the set of potential TALE target genes was delimited as the top scoring 200 genes for each TALE. This set of tentative target genes was then contrasted with experimental expression data. Those targets that showed an expression pattern consistent with that of a true target (i.e. induction in appropriate wild-type strain *vs* mock or corresponding T3SS mutant treatments comparisons, see Table S5) were considered as candidate targets for the corresponding TALE. To define an induction criterion, we first inspected the behavior of all six rice control TALE targets from Table 1 (LOC_Os04g19960, *Os8N3/Xa13/OsSWEET11*, *Os11N3/OsSWEET14*, *OsHen1*, *OsTFX1* and *OSTFIIAy1*) in a set of five relevant microarray data comparisons. In all examined comparisons, these genes displayed very high fold change ratios which, as shown in Figure S1, consistently ranked in the top 100 of the lists of significantly induced genes (log_2_ fold change ≥1, adjusted p-value ≤0.1). We therefore adopted a differential expression rank threshold of 100 which was sufficient to capture all control TALE targets, and applied this threshold to select Talvez predictions exhibiting an induction pattern similar to genuine TALE targets.

Because we were primarily interested in delineating a broad set of high-quality candidate TALE targets, we further investigated, in this genome-wide framework, which position correction parameter value improved our ability to identify induced genes harboring a predicted EBE in their upstream region. To this end, we ran Talvez predictions for all query TALEs (Table S4) with various position correction values (15–20), including no correction. As shown in Table S6, position 19 maximized the total number of genes that were both Talvez-predicted and differentially expressed. It also maximized the number of TALE having at least one candidate target. Importantly, with this position, it was possible to identify 13 out of the 14 pairs identified by the first version of Talent [5] for *Xoo* PXO99^A^ and *Xoc* BLS256 strains (see our resulting list of candidates in Table S7). The missing Tal7a/8a-LOC_Os01g68740 pair [5] was predicted by Talvez but did not meet our criteria for differential expression. Hence, we concluded that examining the differentially expressed top 200 Talvez predictions with a position correction value of 19 produces an exhaustive and high quality perspective on susceptible rice candidate TALE targets.

### The Inferred TALEs-candidate Target Genes Regulatory Network is Potentially Enriched for Functional Hub Genes

**Figure 4:**
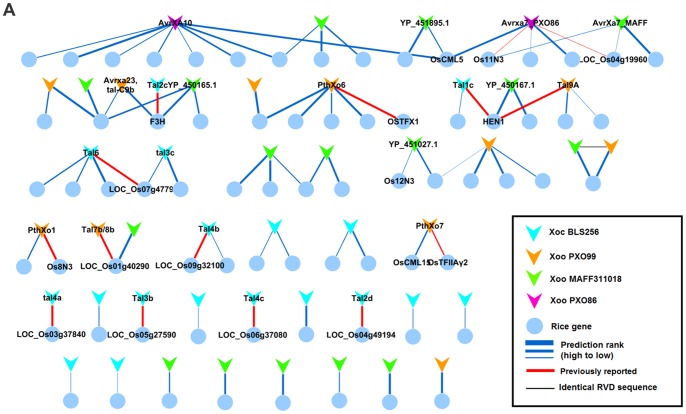
A TALE-candidate target gene network. Hierarchical representation of the TALE-candidate target gene network; genes are represented by circles and TALEs by polygons. Only the names of genes and targets discussed in the main text are shown. Alias or common names for TALEs are shown when available or the GeneBank accession number is given instead. Increasing edge thickness indicates better EBE prediction ranking. Interactions previously reported in the literature are highlighted with red edges.

By comparing Talvez predictions and microarray data with the parameters defined above, a set of 73 rice genes was predicted to be induced by 47 TALEs (Table S7 and Datasets S1). On the other hand, for the remaining 22 TALEs, no differentially expressed target gene was found. The differentially expressed genes and their corresponding TALEs were organized into a TALE-gene network of 91 interactions (Figure 4), 17 of which correspond to our controls or to interactions already predicted in the literature (Table S7). This network contains 17 instances of simple one-to-one interactions between a TALE and a putative target. The remaining interactions are organized into more complex modules corresponding to multiple TALEs from identical or distinct strains predicted to target the same genes or conversely, single TALEs predicted to target multiple genes (Figure 4). The annotated functions in this candidate TALE target gene set are very heterogeneous, and no enrichment of any GO term was found using Singular Enrichment Analysis (SEA) (see Figure S2A) [34]. However, a possible commonality of TALE-target genes is their role in functional networks. Of the 73 candidate targets, 43 genes (58%) were found to be highly connected (degree >5) in a Rice network of predicted functional interactions [35] (Figure S2B). The percentage of highly connected genes among TALE targets was significantly higher than among sets (n = 100) of randomly selected rice genes (Figure S2C). This suggests that TALEs might converge into targeting highly connected hubs in plants networks, as described for *Pseudomonas syringae* type III virulence effectors in Arabidopsis [36].

**Figure 5:**
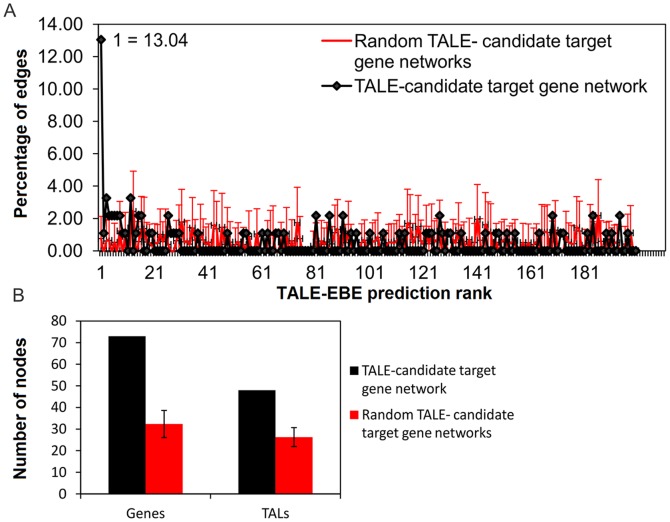
Comparison of the TALE-candidate target gene network with random networks obtained with shuffled TALEs. Properties of the TALE-gene network are compared to average values from 100 randomized controls (error bars indicate standard deviation): (**A**) percent frequency distribution of Talvez prediction ranks of TALE-gene pairs, the percentage of top (#1) ranking TALE-gene pairs is indicated for the TALE-gene network. (**B**) Number of genes and TALEs in the TALE-gene network compared to control random networks.

To assess the possibility that associations between differential expression and EBE predictions are fortuitous, for example due to the prediction of ubiquitous sequences in the genome, a set of 100 random TALE-gene networks was used as a negative control and compared with the original network. These random networks were generated by shuffling the RVD sequences of the TALEs used for the original network then predicting EBE for these shuffled TALEs in the rice genome and filtering the predictions based on microarray data. As a result, the random networks were composed of less elements (TALEs and promoters) and the distribution of the prediction ranks was significantly different from the original network (Kolmogorov-Smirnov test p-value 2.2e-16) showing no prevalence of high ranking genes (Figure 5A and B). This indicates that a large fraction of the edges in the TALE-gene network represents biologically relevant, non-random associations. It is however possible that many of the low ranking predictions (right tail of the distribution in Figure 5A) derive from spurious associations.

### The TALE-candidate Target Gene Network Contains Several Instances of Multi-gene Targeting by Single TALEs

As exemplified by the case of AvrXa7 from strain PXO86 that has been shown to directly induce transcription of both *Os11N3* and LOC_Os04g19960 [14], [16], there is the under-scrutinized possibility that individual TALEs are actually able to coordinately induce plant gene sets. In the network of Figure 4, 23 TALEs (48%) had more than one potential target gene. Yet, no correlation was found between the number of target genes for each TALE (degree) and TALE length (Spearman correlation coefficient = 0.1), nor between TALE degree and specific RVD compositions (Spearman correlation coefficient from −0.2 to 0.4) (Figure S3). This suggests that targeting of multiple host genes may not be attributable to RVD number and composition bias and that individual TALEs may actually control gene sets composed of multiple virulence targets and/or off-targets**.** To support this notion with more discriminatory expression data, we identified Talvez-predicted genes specifically induced by individual TALEs by comparing the expression profiles of plants inoculated with wild-type bacteria and mutants for PthXo1 (GSE36272, PXO99^A^ vs PXO99^A^ ME2) and both PthXo6 and AvrXa27 (GSE36272, PXO99^A^ vs PXO99^A^ ME1). For the PthXo1 experiments, from the top 100 most induced genes, only the known *Os8N3* target had a predicted EBE (Table S8), indicating that this TALE probably does not have additional targets in the rice genome. Therefore, the other predicted target for PthXo1 detected in the PXO99^A^ versus PXO99^A^ T3SS mutant microarray data comparison on Nipponbare (Table S7) is likely a false positive. In contrast, for the PthXo6/AvrXa27 experiment, in addition to the known PthXo6 target *OsTFX1* (LOC_Os09g29820), up to six genes were significantly induced in the wild type versus double mutant strain comparison on at least one rice variety and had a high-scoring Talvez-predicted EBE for PthXo6 (Table S8).

### TALE-candidate Target Gene Networks Reveal within-strain Functional Redundancy and between-strain Functional Convergence as Remarkable Features of TALE Repertoires

**Figure 6:**
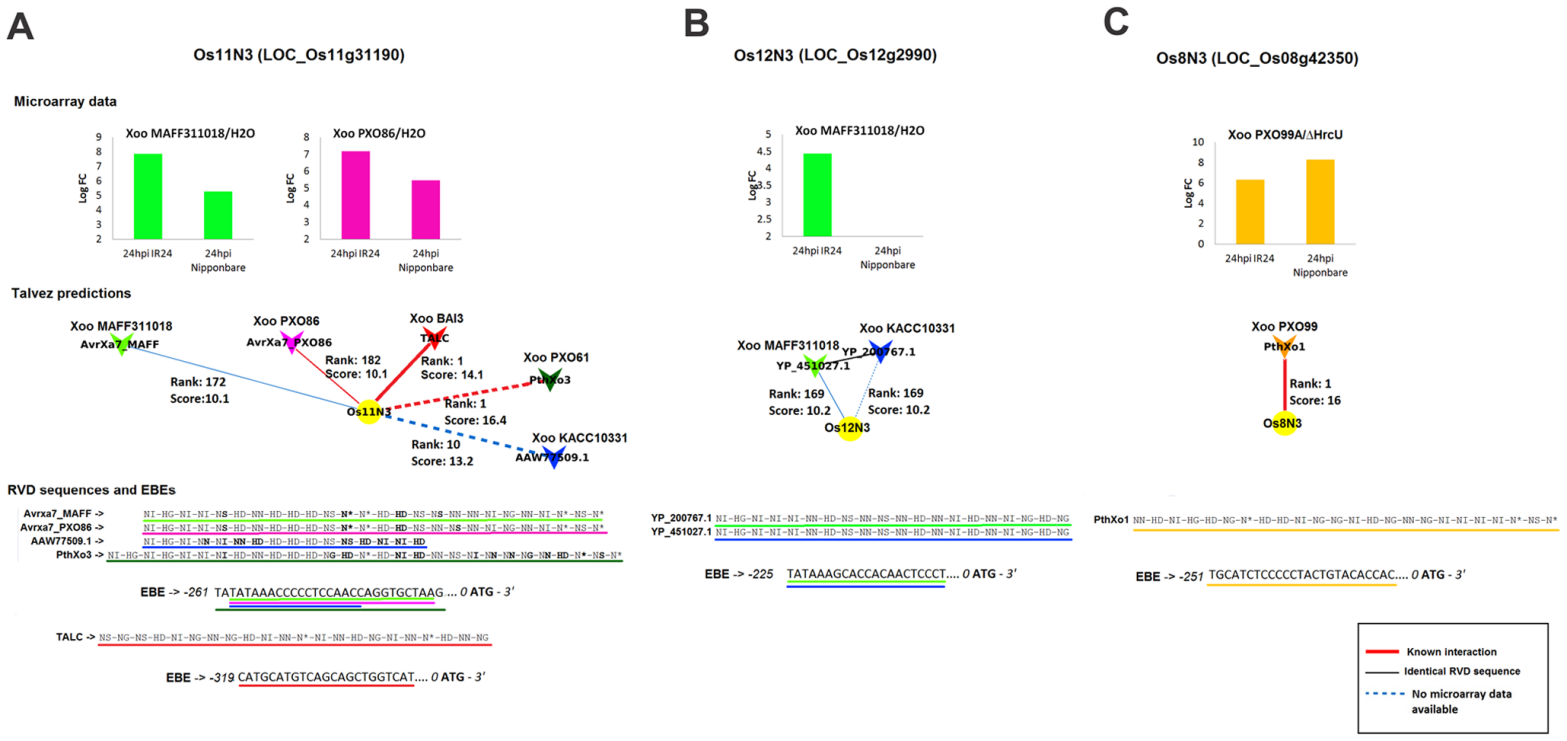
TALEs from multiple *Xoo* strains may converge onto three distinct MtN3 gene family members. Panels (**A**), (**B**) and (**C**) summarize Talvez predictions and expression data respectively for *Os11N3*, *Os12N3 (Xa25)* and *Os8N3* (*Xa13*). From top to bottom: data in bar plots derive from our analysis of microarray data from different rice genotypes and 24 hours after infection time points (hpi). Relevant treatments comparisons are indicated above the graphs. logFC values correspond to log2-transformed fold-change ratios. In the Talvez prediction network snapshots, the rank and score values along the edges represent Talvez prediction output for the connected gene (EBE) in target searches for the corresponding TALE. The bottom part of each panel contains a manual alignment of the RVD sequences from TALEs that are predicted to target the gene under consideration in the panel. Individual residues highlighted in bold deviate from the consensus at that position. The locations of the predicted EBEs on the upstream sequences of the rice gene are marked by lines colored following the same pattern as on the RVD alignment. Numbers on the left indicate the distance in base pair between the most upstream nucleotide of the reported sequence and the ATG. TalC from the African Xoo strain BAI3 which has been reported to target *Os11N3* [18] was included in panel A to illustrate the notion of convergence on gene susceptibility targets at the level of distinct EBEs.

Various TALEs from unrelated *X. oryzae* strains directly induce members of the *MtN3* gene family (also known as *Nodulin-3* or *SWEET*) [16], [18], [32], [37]. This represents a strong case of functional convergence for inducing functionally related genes. Here, besides the known TALE targets *Os8N3* and *Os11N3*, another *MtN3/SWEET* (LOC_Os12g29220) was found to be putatively targeted by a TALE (YP_451027.1) from MAFF311018 (Figure 6). This gene, hereafter referred to as *Os12N3*, is closely related to *Os11N3* and was recently shown to correspond to the recessive resistance gene *xa25* conferring race-specific resistance to the *Xoo* strain PXO339 [38]. Interestingly, in addition to the YP_451027.1 TALE, MAFF311018 also possess an *avrXa7* homolog that is predicted to target *Os11N3* (Figure 6). Altogether these observations raise the possibility that the YP_451027.1 and AvrXa7 TALEs, both encoded by the same strain could act redundantly on distinct *MtN3/SWEET* family members on the IR24 variety. In order to find additional evidence for TALE functional convergence, we ran Talvez predictions for sequenced TALEs from other *X. oryzae* strains for which no expression data are available (29 TALEs, 16 from the fully sequenced strain KACC10331, see Table S4). We then constructed a “conserved target” network by incorporating new TALE nodes into our previous network. These nodes were connected to existing rice genes if these genes where among the 200 best ranking Talvez predictions for these TALEs. As a result, 24 additional TALEs were predicted to bind to the promoter of 33 genes in the network (Figure S4, Table S7 and Datasets S2). Strikingly, analogous to the situation observed for the Japanese strain MAFF311018, two TALEs from the Korean strain KACC10331 were also predicted to target *Os11N3* and *Os12N3* (Figure 6), indicating that internal genetic redundancy among TALEs targeting *MtN3/SWEET* family members may be a recurrent feature of TALE repertoires. Interestingly, LOC_Os12g41110 (*OsCML5*) and LOC_Os05g31620 (*OsCML15*) genes which both encode calmodulin-like proteins may represent another case of convergence towards a gene family (Figure 4 and Table S7). *OsCML5* was predicted to be targeted by AvrXa7 and AvrXa10, and was induced in the IR24 cultivar upon infection by *Xoo* strain PXO86. Similarly, *OsCML15* (LOC_Os05g31620) was induced in Nipponbare inoculated with *Xoo* strain PXO99^A^ versus the T3SS mutant ME7, and contained a predicted EBE for PthXo7. In rice, Calmodulin-like proteins are known to be necessary for colonization of plants by both pathogenic and symbiotic organisms [39], [40].

**Figure 7:**
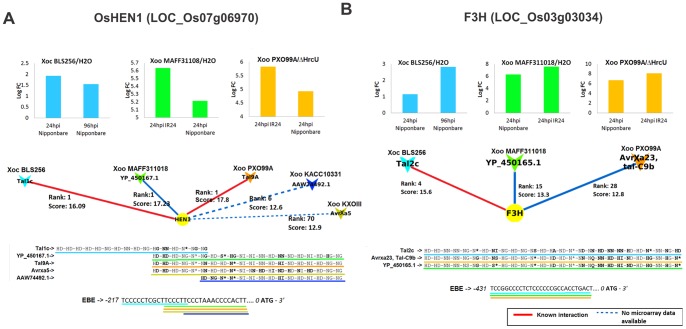
Possible functional convergence on specific rice TALE targets between *X. oryzae* pathovars. Panels (**A**) and (**B**) summarize Talvez predictions and expression data respectively for *OsHen1* and *F3H* (LOC_Os03g03034). See legend of Figure 6 for details.

**Figure 8:**
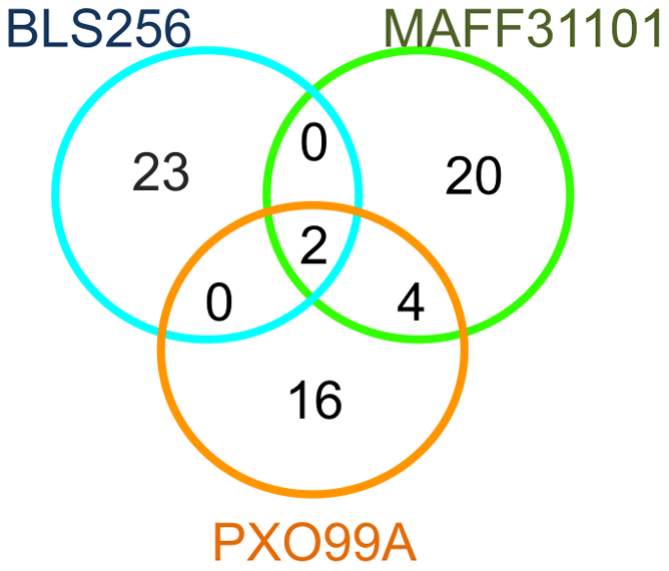
Overlap of candidate rice target gene sets for TALEs from various *X. oryzae* strains. Venn diagram of rice genes from the network assigned to distinct sets according to the strain of origin of their cognate TALE(s).

By analogy with the *Os11N3* precedent [16], [18], those cases in which multiple TALEs target the same gene in non-identical EBEs can be interpreted as evidence of independent adaptative convergence. It is also a strong indication that a candidate TALE target acts as a genuine plant susceptibility factor. In the network of Figure 4, 13 genes (18%) were targeted by more than one TALE (with distinct RVD sequences), and this number reaches 37 (50%) when taking into account the TALEs in the “conserved” network (Figure S4). In addition to *Os11N3*, five genes were found to be targeted by multiple TALEs in distinct EBEs (LOC_Os11g26790, LOC_Os01g51040, LOC_Os05g11840, LOC_Os07g47790, LOC_Os07g06970). They therefore represent prime candidates for novel plant susceptibility factors. One of these loci corresponds to *OsHen1* (LOC_Os07g06970) which is probably targeted by the *Xoc* TALE Tal1c from BLS256 and the *Xoo* TALE Tal9A from PXO99^A^ [5]. Here, we predict that it may also be targeted by one TALE from MAFF311018, as well as TALEs from strains JXOIII and KACC1033 (the last two without microarray support) (Figure 7A). Curiously, the TALE from JXOIII targeting *OsHen1* was recently claimed to be the avirulence factor *avrXa5* [41]. Apart from *OsHen1*, the only other apparent case of convergence between *Xoc* and *Xoo* strains (Figure 8 and Figure 7B) is the LOC_Os03g03034 gene, a predicted flavanone 3-hydroxylase (F3H). This F3H-encoding gene is likely induced by AvrXa23 from PXO99^A^, Tal2c from *Xoc* strain BLS256 (also reported in [5]) and YP_450165.1 from *Xoo* strain MAFF31101, albeit through an identical EBE (Figure 7B). Members of this gene family are involved in the synthesis of flavonoids, which are known to play a role in plant-bacteria interactions, mainly in defense against pathogens and communication with beneficial symbionts [42]. Other interesting potential cases of functional convergence both between and within strains, as shown in Figure 4, were those of LOC_Os11g26790 and LOC_Os07g47790. LOC_Os11g26790, a dehydrin gene, is predicted to have two different EBEs for two TALEs from PXO99^A^ as well as for two TALEs from MAFF311018. Likewise, LOC_Os07g47790 encodes a putative AP2 domain-containing protein and is predicted to have two distinct EBEs for two different TALEs from *Xoc* BLS256: Tal3c and Tal6 (See also Table S7). Interestingly, both gene families have been implicated in plant biotic and abiotic stress responses [43], [44]. Altogether, these findings strengthen the idea that functional redundancy is a major organizing principle shaping TALE repertoires composition both between and within strains.

## Discussion

One major output of this work is the design of a more accurate prediction tool for TALE binding sites in DNA sequences. In conjunction with public expression data, this advantage was exploited to elucidate putative targets and potential functional interactions among TALE repertoires of four bacterial strains yielding an original repertoire-wide perspective on the virulence strategies of rice-infecting *X. oryzae* strains.

### Performance of Prediction Programs and Features of TALE-EBE Specificity

We initially tested the performance of three programs for EBE prediction: Talvez, the program that performed the best, is based on transforming TALEs RVD sequences into PWMs and then scoring all possible binding sites in a promoter region using a log-likelihood function. Storyteller, another program developed in this work, took advantage of a faster pattern-search algorithm based on Hidden Markov models. While both programs had similar rates of true and false positives in the validation set, when searching the complete set of rice promoters Storyteller ranked control targets lower than Talvez. We are currently trying to incorporate additional corrections that may improve Storyteller’s predictions while keeping its main advantage: greater speed. The search strategy used by Talvez is similar to the one used by the TALE target finder from the Tale-nt suite [23]. To a large extent, the differences in performance between the two programs are likely due to the use of different RVD-nucleotide specificities and different scoring functions where Talvez takes into account the background distribution of nucleotides in the promoter regions.

A detailed look into characterized RVD-DNA interactions indicated that perfect RVD-nucleotide pairing in TALEs N-terminal region (first 15–19 RVDs) probably determines for the most part the target DNA recognition and activity. In contrast, downstream positions seem to hold little information for identifying legitimate EBEs. In agreement with this, Kay et al. [20] introduced mutations in the last three positions of the AvrBs3 binding site UPA20 that had no effect on TALE activity. Conversely, the sequence of the AvrBs4 TALE EBE of *Bs4C*, an executor type resistance gene, exhibits a simple 2-bp polymorphism located at 5′ positions 3–4 (Table S2) which explains why the susceptible allele is unable to recognize AvrBs4 [30]. Currently, the most convincing experimental evidence in favor of such a polar effect in TALE-DNA recognition is probably the recent work by Meckler et al. [45]. Using both *in vitro* affinity and *in vivo* activity assays, they demonstrated that N-terminal RVDs contribute more to the overall DNA affinity than C-terminal repeats and that mismatches at the 3′-end of the target DNA are more tolerated than at its 5′-end. They note, however, that this effect is more pronounced with artificial TALEs than with the natural AvrBs3 TALE. These findings let them introduce an “N-terminal organizing center” hypothesis that tries to explain why positions after the 15th RVD may still contribute to activity in specific contexts.

The control targets of four TALEs, namely AvrXa7, PthXo3, AvrXa27 and Hax2, were hard to predict with any of the programs and ranked very low in whole genome searches. AvrXa7 and PthXo3 are particularly long TALEs with a high number of RVD-nucleotide mismatches in the DNA-binding sequence upstream of their respective target. The predictions for these TALEs were however largely improved when a position correction was applied to Talvez. In contrast, AvrXa27 and Hax2 retained relatively low rankings, despite a slight improvement in the detection of their respective EBEs upon applying the position correction. These TALE-EBE pairs probably correspond to atypical interactions that cannot be explained solely on the basis of the standard rules of RVD-nucleotide association. Clearly, the current model of TALE-EBE specificity is not perfect and does not yet account for less characterized or unsuspected aspects of TALE-DNA interactions. For example, it was recently demonstrated that different RVDs have different contributions to DNA binding [45], [46]. A more detailed knowledge of the contributions of individual RVDs to affinity and how these contributions are distributed and interact along the DNA target sequence may eventually be useful to improve predictions for TALE-EBE pairs such as the AvrXa27-*Xa27* (LOC_Os06g39810) and Hax2-*PAP1* (AT1G56650). Our preliminary attempts to incorporate a matrix with higher scores for strong RVDs yielded no prediction improvement in our validation set (data not shown). Another feature that could improve predictions is a better knowledge about the effect of the genomic context of the EBE on TALE binding or activity (e.g. distance to TATA box, distance to translational start sites). Yet, this aspect remains poorly explored.

### *X. oryzae* Genomes-wide Candidate TALE Targets Mining: One TALE, Several Targets?

Because of the key role of T3SS substrates in the infection process, identification of type III effector host targets may be the fastest route to the molecular dissection of virulence strategies of pathogenic bacteria relying on such systems, and on the host biological processes that are perturbed to create disease-promoting conditions. In this respect, TALEs are interesting type III effectors because they manipulate the host physiology by direct transcriptional up-regulation of susceptibility genes. Hence, the ability to accurately predict TALE-binding elements in rice promoters enables a straightforward comparative perspective on individual TALEs targets as well as on the organization of TALE repertoires: how they are functionally structured with respect to their virulence target genes and how these repertoires may evolve. The performance of Talvez allowed a systemic insight into the sets of candidate target genes of *X. oryzae* strains. Putative targets for a total of 69 TALE sequences from four strains were identified in the *O.sativa* japonica genome using a combination of software predictions and expression data analysis. On top of this high-quality TALE-candidate rice target gene network, Talvez predictions for 29 additional TALEs identified genes that may be repeatedly targeted by *X. oryzae* strains.

While it has been previously reported that individual TALEs can induce the expression of multiple genes [14], [20], our analysis of TALE-target gene networks suggests that the extent at which this may occur is striking (∼50% of TALEs in the network had multiple targets). This observation raises the question of the functional interplay between genes within individual sets, as well as the biological relevance of these sets of co-regulated genes in the parasitic process. Many of the “additional” targets for each TALE would likely have no physiological effect and may be the result of fortuitous, adaptatively neutral pairings as it seems to be the case for AvrXa7 from *Xoo* PXO86 and its secondary target LOC_Os04g19960 [14] which is annotated as a retrotransposon protein. These findings furthermore underscore the importance of carefully considering the probabilities of off-targets when TALEs are to be applied to biotechnological engineering. Here, Talvez is expected to improve the design of artificial TALEs.

On a contrasting note, it is worth pointing out that our microarray-supported Talvez EBE predictions did not identify any targets in the Nipponbare genome for 22 out of the 69 TALEs in the query set. This apparent absence of candidate targets might stem from an intrinsic inability of our algorithm to predict accurate targets for this specific set of TALEs as discussed above. Relevant EBEs for these TALEs may also have been missed because they are located outside of the 500-bp upstream regions of annotated objects represented in the Affymetrix chip. On the other hand, it is likely that cognate EBEs are genuinely absent from the Nipponbare genome and that searching other genomes, e.g. from indica varieties, may ultimately identify true targets. Alternatively, as proposed by Yang and Gabriel [47], these TALEs may not have a relevant target at all and may be maintained as a reservoir for the evolution of new TALE-target specificities via recombination among TALE genes, thus facilitating adaptation to changing host genotypes.

### Universal Virulence Targets of *X. oryzae* TALE Repertoires: An Emerging Model

Our comparative analysis of the sets of rice genes potentially targeted by the complete TALE repertoires from *Xoo* and *Xoc* strains provides a novel perspective on genes or gene families that repeatedly appear to be induced by TALEs. The observed overlap of our high-quality rice candidate target sets across strains is relatively limited and rather unexpected, especially for the PXO99^A^ and MAFF311018 strains which belong to the same *Xoo* pathovar but share only six common targets (Figure 8). One interpretation of this situation is that the contribution of TALE repertoires to a pathogenic lifestyle is achieved through the induction of mainly unrelated primary target gene sets that however converge functionally on common processes. It is also possible that induction of only a few conserved targets is key to sustain a rice parasitic lifestyle and that the induction of others is accidental and makes no contribution to plant tissue colonization. Out of the 77 genes in the network, *OsHen1* (LOC_Os07g06970) stands out as being putatively targeted by 5 distinct TALEs from all *Xoo* and *Xoc* strains with a complete TALE inventory, plus the alleged [41] AvrXa5 avirulence protein. This observation suggests that this putative methyltransferase, a central component of small RNAs biogenesis pathways [48], may be universally targeted by all *X. oryzae* strains regardless of their respective tissue tropism but a role of *OsHen1* as a susceptibility gene has not been described yet. Similarly, LOC_Os03g03034 annotated as a flavanone 3-hydroxylase (F3H) with potential roles in plant-bacteria interactions could belong to the same universal target category. However, no cognate TALE was found for this F3H in the KACC10331 genome. In rice, F3H proteins have been found to be induced in multiple resistant interactions [49]. Whether the induction of F3H family members by these TALEs contributes to resistance and/or virulence is intriguing and will be addressed in future studies.

Remarkably, our work and earlier studies of others provide strong evidence that induction of a member of the MtN3/SWEET family, presumably as a way to extort carbon sources from the host, is an absolute requirement for *Xoo* strains to successfully colonize rice xylem vessels. Indeed, it was previously shown that three unrelated *Xoo* strains require TALE-mediated transcriptional induction of a MtN3/SWEET family member for full virulence on susceptible rice varieties [16]–[18]. Our “conserved” network further extend these results by identifying additional TALEs from MAFF311018 and KACC10331 strains that are predicted to up-regulate *Os12N3,* another member of the MtN3/SWEET family which corresponds to the responsive allele (*Xa25*) of the *xa25* resistance gene [38]. The *Os12N3* susceptibility allele (*Xa25*) is induced by PXO339 in susceptible varieties, including Nipponbare [38]. Its induction by artificial TALEs was recently shown to increase susceptibility to Xoo strains [50]. Moreover, the promoter region of *Os12N3* shows various polymorphic sites possibly associated with resistance (including two polymorphic sites in the predicted EBE for YP_451027.1) [38]. Thus PXO399 most probably expresses a TALE that specifically recognizes the promoter region of *Os12N3*. Interestingly, based on our predictions for TALEs of *Xoc* strain BLS256 and an earlier genetic screen with BLS303 [51] which failed to identify a *Xoc* TALE targeting a MtN3/SWEET family member, *Xoc* strains may not require relocation of sucrose to the apoplast or may rely on other sugar efflux mechanisms for infection of the leaf mesophyll. It is noteworthy however that heterologous TALEs targeting MtN3/SWEET family members do confer an increased virulence to *Xoc* [52]. Even if we were unable to demonstrate a specific functional enrichment in our rice candidate target set, it is interesting to note that beyond sugars, TALEs may have evolved to manipulate additional transmembrane transport mechanisms because several candidate targets have an annotation suggestive of a role in ions (sulfate and potassium) and purine derivatives transport. Apart from the MtN3/SWEET family, *OsTFX1* (LOC_Os09g29820, the PthXo6 target) has also been reported to be “universally” induced by *Xoo* strains from diverse geographical origins [19]. In the network, MAFF311018 is the only strain with a complete TALE repertoire that is not predicted to target *OsTFX1*. However, this gene ranks in the 100 most induced genes 24 hours post inoculation with this strain (data not shown), revealing a possible false negative prediction or pointing to an erroneous TALE DNA sequence in databases.

### Functional Redundancy of TALE Repertoires may have Important Consequences on Strategies for Disease Control Relying on Host Genetic Resistance

Another original feature of *X. oryzae* TALE repertoires that emerges from our study is an apparent tendency of individual repertoires to maintain several TALEs capable of redundantly targeting the same or functionally equivalent genes. This seems to be true for the plethora of TALEs in the *Xoc* BLS256 genome, but perhaps the most edifying example of functional convergence involves again the *MtN3/SWEET* gene family. We discovered that strains MAFF311018 and most likely KACC10331 possess a couple of RVD sequence-unrelated but functionally redundant TALEs, each putatively capable of inducing a separate *MtN3/SWEET* gene (Figure 6). It is also likely that analogous to MAFF311018 and KACC10331, a third *Xoo* strain, PXO71, possesses redundant TALEs targeting *MtN3/SWEET* genes because two distinct TALEs from this strain were shown to be able to complement a PXO99^A^ ME2 mutant [51], now known to be defective for *Os8N3* induction [17]. Our assertion that various *Xoo* strains have acquired a redundant set of *MtN3/SWEET*-targeting TALEs is reminiscent of a well described property of *P. syringae* type III effector repertoires that appear to incorporate some degree of functional redundancy among effectors. This feature presumably confers an enhanced robustness to effector repertoires by allowing the costless loss of an effector upon detection by the plant immune surveillance system [53]. One immediate conjuncture of this model applied to TALEs is that contrary to the findings with strains BAI3, PXO99^A^ and PXO86 [18], [19], [54], a single mutation in any of the redundant TALEs in MAFF311018, KACC10331 and possibly PXO71 strains will most likely have few consequences whereas cumulative inactivation of both TALEs should significantly compromise the virulence of the mutant strains. A further ramification of this model in the context of disease control strategies based on varietal resistance is that, in principle, strains like MAFF311018 would overcome resistances based on single loss-of-TALE-responsiveness alleles such as *xa13* (the resistance allele of *Os8N3*), *xa25* (the resistance allele of *Os12N3*), or the ones engineered in the *Os11N3* gene promoter [14]. Considering this aspect and the apparently universal requirement of *Xoo* on *MtN3/SWEET* susceptibility genes to cause disease, it is therefore advisable to pyramide several unresponsive *MtN3/SWEET* genes alleles in a single genetic background, thus likely conferring broad spectrum resistance to bacterial leaf blight. It is important to note however that this strategy could be defeated by strains that have acquired TALEs recognizing novel EBEs, such as TalC for *Os11N3* [18].

In conclusion, this work provides a more accurate tool for mining TALEs virulence targets in the genomes of host plants, as performed here for *O. sativa*, and for predicting artificial TALEs off-target binding sites in biotechnological applications. Our understanding of the global contribution of TALEs to pathogenicity in the *X. oryzae*–rice pathosystem will benefit from the identification of conserved plant targets which tend to be highly connected hubs in functional networks and which may play a major role in susceptibility. The identification of redundant TALEs within individual effector repertoires provides guidelines for their inactivation and for breeding and engineering resistant plant varieties. In the future, Talvez in conjunction with gene expression data will greatly facilitate the identification of candidate TALE-dependant immunity loci in the genomes of resistant accessions in the germplasm of major crops.

## Materials and Methods

### Talvez Description

Talvez is implemented in the Perl and Java programming languages and is based in part on a method for transcription factor binding sites identification relying on Positional Weight Matrices (PWM) that shows great accuracy when accompanied with a log-likelihood scoring system taking into account nucleotide distributions in scanned sequences [24]. Talvez scans DNA sequences for EBEs in several steps: (i) for each input TALE RVD sequence, a PWM is created according to the RVD-nucleotide association matrixes described below (Table S1). If the position correction is specified, the scaled-down specificities are applied starting after the input position. A user-defined number of pseudocounts can be added to the PWMs, these pseudocounts correspond to a probability value (default = 0.001) added to all instances in the PWM so that any base is allowed to bind at any position albeit with a low probability. (ii) The nucleotide compositions of input DNA sequences (e.g. full genome sequences or promoter regions) are calculated. (iii) Input DNA sequences are screened and scored using a log-likelihood function provided by the Java script available from [24] that weights the probability of a nucleotide corresponding to the PWM vs the probabilities of finding this nucleotide in any position according to the nucleotide composition. (iv) Results above a user-defined threshold value are reported and ranked according to their score. The program scans only the forward strand of the sequences and does not allow gaps. The following parameters can be modified: the RVD-nucleotide association matrixes, the RVD after which the position correction is applied, the threshold score to report results, the number of pseudocounts, and the total number of results to report. The web version (http://bioinfo.mpl.ird.fr/cgi-bin/talvez/talvez.cgi) allows scanning of 25 preloaded complete promoters sets from plant genomes such as *O. sativa*, *A. thaliana*, *Vitis vinifera* or *Populus trichocarpa* downloaded from Phytozome (http://www.phytozome.net/).

### Storyteller Description

Storyteller was written in the Perl programming language. It relies on hidden Markov models and works as follows: (i) The program converts a TALE RVD sequence into a PWM and uses this matrix to generate a set of possible binding sequences (rounds). (ii) While generating the sequences, an error function introduces “noise” to allow the creation of mismatching sequences. The noise is reflected in the probability of generating any nucleotide for a possible binding sequence regardless of the RVD specificities. The probability can be zero, if the error function is set to “none”, or kept constant if the error function is set to “constant”. The noise can also be set to depend on the position. It can either linearly increase or decrease along the sequence if the error function is set to “linear”, or it can be set to “parabolic” to allow more noise at both ends, or it can be “Hvaa or RVD dependent” resulting in less noise for the best characterized RVDs. In addition to the noise function, the PWM also allows the use of pseudocounts. (iii) The generated sequences are converted into a HMM using hmmbuild from the HMMER2 software [55]. (iv) The model is used to scan the genomic regions using hmmsearch from HMMER2 [55]. hmmsearch allows to include gaps and to specify a minimum score or e-value as threshold to report a candidate match.

### RVD-nucleotide Association Matrixes

To encode RVD-nucleotide association specificities, the values of the counts matrix originally assembled by Moscou and Bogdanove [5] were adjusted in order to incorporate more recent findings and a priori assumptions in the association model: (i) equal counts vectors were assigned to RVDs ending with the same amino acid, keeping those of the most frequent RVD; (ii) specificities for the 0 position were modified to allow the binding of cytosine, albeit with a lower count than thymine. The resulting matrix (reported in Table S1) is scaled similarly as originally reported [5], resulting in a greater contribution to the overall prediction score for better characterized RVD, such as and HD, NI, NN, NG. In addition, a few specificities were also refined by trial-and-error on the validation set. The scaled down RVD-nucleotide association matrix for the position correction (Table S3) was constructed by assigning the same value for all preferred DNA base matches (darkest grey cells in Table S1) and the same, lower value, for all non-preferred matches. This matrix was adopted after preliminary tests against other possible options like not scoring matches at all after a certain position.

### EBE Prediction Input Parameters

Predictions for Talvez used the following parameters: pseudocounts ‘1e-05’, minimum score ‘9’, number of reported TALEs ‘all’ and various position corrections. Predictions for Storyteller used the following parameters: rounds ‘1e5’, noise ‘0.5’, noise-shape ‘hvaa-dependent’, max e-value ‘700’, minscore ‘2’, gap probability ‘1e-3’. Predictions for Tale-NT [23] were run on the web-server with default parameters, screening against the Arabidopsis or Rice sets of predicted promoters and allowing binding of C or T at position 0. Results were filtered to include only genes with predicted boxes at a distance of less than 500 bp from the ATG translational start codon and scores were scaled x^−1^ to allow comparisons with the other programs. EBE screenings were made against the promoter regions (500 bp upstream of the translation start site) of predicted genes in the *A. thaliana* (TAIR v 10) and *O. sativa* (MSU 7.0). Promoter sequences were extracted using the BioMart tool at www.phytozome.com [56]. Prior to scanning the Nipponbare MSU 7 genome, the binding box from the unrecognized xa27 allele was substituted with the binding box of the Xa27 responsive allele [57].

### TALEs Queries and the Validation Set of TALE-DNA Pairs

A comprehensive validation set of negative and positive interactions was build based on published TALE-DNA pairs that have been tested experimentally by GUS assays, electrophoretic mobility shift assay and/or QRT-PCR [4], [5], [14], [16]–[21], [26]–[30] (Table S2). Interactions that exhibited detectable induction in an experiment and that were interpreted as positive in the original sources were defined as “positive”. In two cases (PthXo7- OsTFX1ΔT, Avrxa7- Os11N3ΔT), significantly reduced induction activities were reported [26], these interactions were nonetheless considered positive because of their detectable activity in a GUS-assay and because the corresponding mutations (T to C at position 0) are known to be functional. Negative interactions consisted of mutated boxes of otherwise positive interactions that showed no induction activity in the experiments. Note that the interactions involving AvrBs4 and Bs4C (blue background in Table S2) were not included in the analysis reported in Figure 1, Figure 2 and Figure 3A. A subset of this validation set comprising known natural targets for TALE in the rice and *Arabidopsis* genome was used for whole-genome screenings (Table 1, Table S2). RVD sequences for TALEs in the validation set and for all other known TALEs referenced at the Xanthomonas Resource Database (http://www.xanthomonas.org/, http://bioinfo-prod.mpl.ird.fr/xantho/x.org/gui/) were manually extracted from the available protein sequences at NCBI. Sequences from pseudogenes or TALEs with less than two RVDs were not included. When two database entries corresponded to the same RVD sequence in the same strain only one entry was kept. All RVD sequences for TALEs in our validation set were confirmed with those in the corresponding literature. These sequences as well as other information relative to the TALEs included in the analysis (e.g. GenBank accession numbers, common name, strain of origin) can be found in Table S4.

### Receiver Operating Characteristics (ROC) Analyses

The outputs of the prediction softwares obtained after screening the validation set of experimentally tested positive and negative interactions were analyzed as follows: for each TALE and each program, a minimum classifying threshold was defined as the lowest score for a genuine positive EBE, all EBEs scored above or equal to that threshold were considered positive predictions and all EBEs scored below that threshold were considered negative predictions. In this way a true positive corresponded to a genuine positive EBE scored equal or above the minimum threshold (i.e. scored higher than all negative), and a false positive corresponded to a genuine negative EBE scored equal or above the minimum threshold (i.e. scored higher or equal than a known positive). For construction of the ROC graphs, the true and false positive rates were calculated as specified in [58].

### Microarray Analyses and TALE–candidate Target Network Construction

Routine computational tasks were performed in R with facilities provided by packages from the Bioconductor project [59]. Primary expression data used for gene differential expression analysis corresponds to the following Gene Expression Omnibus (GEO) datasets: “Comparative transcriptional profiling of rice undergoing infection by *X. oryzae* pv. *oryzae* or by *X. oryzae* pv. *oryzicola*” (GSE16793), “Comparison of transcriptional responses of two susceptible rice cultivars to strains of *Xanthomonas oryzae*” (GSE36272). The Affymetrix probe-level expression data in CEL files was processed separately for each dataset using the Bioconductor affy package [60] with default parameters of the rma function that computes the RMA (Robust Multichip Average) expression measure after background correction, normalization and probe summarization. To identify differentially expressed genes at a given time point after inoculation on susceptible plant cultivars, pair-wise comparisons that tested (i) a wild-type strain versus mock treatment (virulent strain effect), (ii) a wild-type strain versus a T3SS-defective mutant strain (T3SS effect) or (iii) a wild-type strain versus a mutant strain in a TALE gene (specific TALE effect) (Table S5) were performed using the Bioconductor limma package as described in [61]. The probesets that displayed a value of the log_2_-transformed fold change (logFC) equal or above 1 and an associated adjusted p-value (Benjamini and Hochberg’s adjustment method) equal or below 0.1 in a treatment comparison were deemed as differentially expressed. Their expression data (logFC and logFC rank) and associated limma statistics in that comparison were recorded in a custom sqlite database. This database also stored the Talvez predictions on the corresponding 500 bp promoters set and was queried to retrieve TALE targets satisfying specific criteria as described in the results section. The mapping of Affymetrix probe sets on MSU6 Gene Model IDs was obtained from the www.ricechip.org website.

Random TALEs – rice genes networks were constructed by first generating six random TALEs for each natural *X. oryzae* TALE encoded in PXO99^A^, MAFF311018, PXO86 and BLS256 strains by shuffling their RVD sequence. Next, 100 random networks were assembled iteratively by randomly selecting one of the six shuffled TALE derivative for each initial natural TALE and querying the database for its associated Talvez-predicted targets that also met the microarray expression criteria. The distributions of node properties such as Talvez rank and connectivity measures (TALE and rice gene degrees) were computed in R with facilities from the graph package of Bioconductor.

### Additional Analyses

Perfect matches (PMs) in the validation set of positive and negative interactions were identified using an in-house Perl script that identified PMs in each position based on the RVD-nucleotide specificities used by Talvez. Connectivity of TALE target or random gene sets in was assessed by querying the Ricenet [35] server online (http://www.functionalnet.org/ricenet/search.html) and retrieving the complete output network (query and all predictions). TALE-gene network and functional networks for TALE targets obtained from RiceNet were visualized using Cytoscape [62]. Singular Enrichment Analyses (SEA) for GO terms were performed on the AgriGO platform [34] against the *O. sativa* MSU7 nonTE background and by testing with Fisher and hypergeometric methods at 0.05 significance. Other advanced options were left unchanged. Statistical tests for distribution and mean comparisons were performed in R.

### Predictor Programs Availability

Packages containing the source code of the Talvez and Storyteller programs are available in Datasets S3 and Datasets S4, respectively. A web interface and the source code of the Talvez and Storyteller programs are also accessible on our server at http://bioinfo.mpl.ird.fr/cgi-bin/talvez/talvez.cgi, and http://bioinfo-prod.mpl.ird.fr/xantho/tales, respectively. Finally, the source code of the Talvez and Storyteller is also available for download on Sourceforge at https://sourceforge.net/projects/talvez/and https://sourceforge.net/projects/storytellr/respectively.

## Supporting Information

Figure S1 **Distribution of the relative expression ranks of control TALE target genes in microarray data.** The x-axis values correspond to a decimal log transform of the rank of the log2 fold change (logFCRank) of the Affymetrix probeset(s) corresponding to a control gene in a specific treatment comparison. For all bacterial strain-rice cultivar combinations (see legend), the logFCRank data correspond to a wild type strain versus mock comparison. For PXO99^A^, a wild type strain versus mutant strain defective for type III secretion comparison was also included.
(TIF)

Figure S2 **Relevance of candidate TALE target genes in available rice functional prediction data.** (**A**) Singular Enrichment Analyses for GO terms associated with candidate TALE target genes (input list) compared to background annotations in rice performed with AgriGO. No significant differences were found (Fisher exact test, p-value <0.05). (**B**) Predicted functional network for candidate TALE target genes in the probabilistic functional gene network RiceNet. Candidate TALE target genes are shown in red, non-TALE targets are shown in green (C) Frequency of highly connected genes in RiceNet among candidate TALE targets compared to randomly selected sets of rice genes.
(TIF)

Figure S3 **A survey of possible association between in-network TALE degree and TALE RVD sequence features.** Dispersion plots showing the relation between TALE degree (total number of targets) and TALE length (in number of repetitions) or TALE common RVDs frequency (percentage) in the predicted TALE-target gene network. Spearman correlation values are shown between parentheses.
(TIF)

Figure S4 **Representation of the “conserved target” network.** See the legend of Figure 4A for details. Gray color represent Avrxa7-related TALEs from Xoo strains: KXO85, PXO0314, PXO2648, PXO348, PXO356, PXO357, PXO557 and an in-vitro Avrxa7 mutant.
(TIF)

Table S1 Updated RVD-nucleotide association matrix used by Storyteller and Talvez to produce PWMs. Darker gray shading indicates increasing binding preferences for each RVD. Higher counts were assigned to well-studied RVDs (eg. HD, NG).
(XLSX)

Table S2 Validation set of controls used to test EBE prediction programs. RVDs and promoter sequences were extracted from the indicated references. Interaction class indicate the experimental results,+(positive) = the TALE binds to the EBE or is able to induce the expression of a gene located downstream of the EBE, − (negative) = the TALE is unable to induce the expression of a gene located downstream of the EBE. Raw prediction scores from each program are reported. Each prediction is classified as a true positive (TP), true negative, (TN), false positive (FP) and false negative (FN). Xca = *X. campestis* pv. *armoriaceae*, Xcv = *X. campestris* pv. *vesicatoria*, Xg = *X. gardnerii*, Xoc = *X. oryzae* pv. *oryzicola*, Xoo = *X. oryzae* pv. o*ryzae*. The subset of interactions that were used for genomic screenings and that correspond to genuine targets in the rice or Arabidopsis genome are highlighted in green and underlined.
(XLSX)

Table S3 Scaled-down RVD-nucleotide association matrix. This matrix is used by Talvez to produce PWMs after a specified position when the position correction is applied**.** All perfect matches were assigned a value of 1.5 and all non-preferred matches a value of 1.0.
(XLSX)

Table S4 Supplementary information on TALEs from *X.oryzae* strains used in this work.
(XLSX)

Table S5 Description of the microarray data comparisons performed to identify TALE-regulated genes. Each row corresponds to a pair-wise treatment comparison used in this work to calculate Log Fold changes and select up-regulated genes. Information about the host plant genotype, the query strain, the appropriate reference treatment and the sampling time is provided. Number of TALEs indicates the potential number of TALEs that are expected to be queried for potential rice gene induction activity in that comparison (i.e. difference in TALE numbers between the tested strain and the control treatment). Accession numbers correspond to NCBI GEO Datasets accession numbers.
(XLSX)

Table S6 Assessment of various position correction values for predicting induced TALE targets. Performance indicators for the capacity of Talvez to predict differentially expressed genes (as determined by microarray data) for all sequenced *X. oryzae* TALs when varying the value of the position correction. A gene within the top 200 predicted genes and that also shows differential expression in microarray data is considered as a TALE target gene. Predictions were made against the set of rice promoters for the set of 69 TALE RVD sequences encoded in the genome of strains that have supporting rice expression data. The Number of top or high ranking genes columns reports the cumulated counts of TALE-wise target gene predictions that rank respectively first or in the top 10 in the entire predictions set and that are also differentially expressed. The Sum column displays the row-wise sum of performance indicators values as a composite aggregator value to be maximized.
(XLSX)

Table S7 TALE-candidate target gene network data. Talvez predictions obtained with a position correction value of 19 and cross-referenced with microarray data are reported. Accession number corresponds to the TALE protein sequence available in public databases. RVD sequences were manually extracted from available protein sequences. TALE alias corresponds to a known common name for a given TALE. TARGET gene ID refers to the locus ID of genes containing a Talvez-predicted EBE in their promoters. Prediction scores and rank correspond to the Talvez predictions for the TALE-EBE pair after searching the rice promoters set. Evidence indicates whether the TALE-gene association is supported by gene-induction data in microarrays or if it corresponds to a TALE from a strain without associated microarray data that has a predicted target that is likely induced by TALE from our reference strains (“Conserved target”). The logFC, logFCRank, AveExpr, adj_P_Val and B columns contains respectively the log2-fold change, the rank of the logFC when the values for that comparison (described in the Treatment comparison column) are sorted in a decreasing order, the average log2-expression level for that gene across all the arrays in the experiment, the associated p-value after adjustment for multiple testing and the log-odds that the corresponding gene is differentially expressed in the comparison.
(XLSX)

Table S8 Predicted EBEs in the promoter of differentially expressed genes in microarray comparisons addressing TALE-specific effects. LogFC values correspond to the log2 fold-change from comparisons between plants inoculated with either the wild-type or a TALE-mutant bacteri. EBE distances refer to the translation start site.
(XLSX)

Datasets S1 **Cytoscape Session (.cys) file containing the primary, expression data supported TALE-rice gene network that served for the construction of** **Figure 4A** **.**
(CYS)

Datasets S2 **Cytoscape Session (.cys) file containing the conserved TALE-rice gene network that served for the construction of Figure S4.**
(CYS)

Datasets S3 **Script files for Talvez.**
(TAR)

Datasets S4 **Script files for Storyteller.**
(TAR)
