# Supplementary figures and images for: An Improved Method for TAL Effectors DNA-Binding Sites Prediction Reveals Functional Convergence in TAL Repertoires of Xanthomonas oryzae Strains
Source: PLoS One. 2013 Jul 15;8(7):e68464. doi: 10.1371/journal.pone.0068464 (PMC3711819; doi:10.1371/journal.pone.0068464)

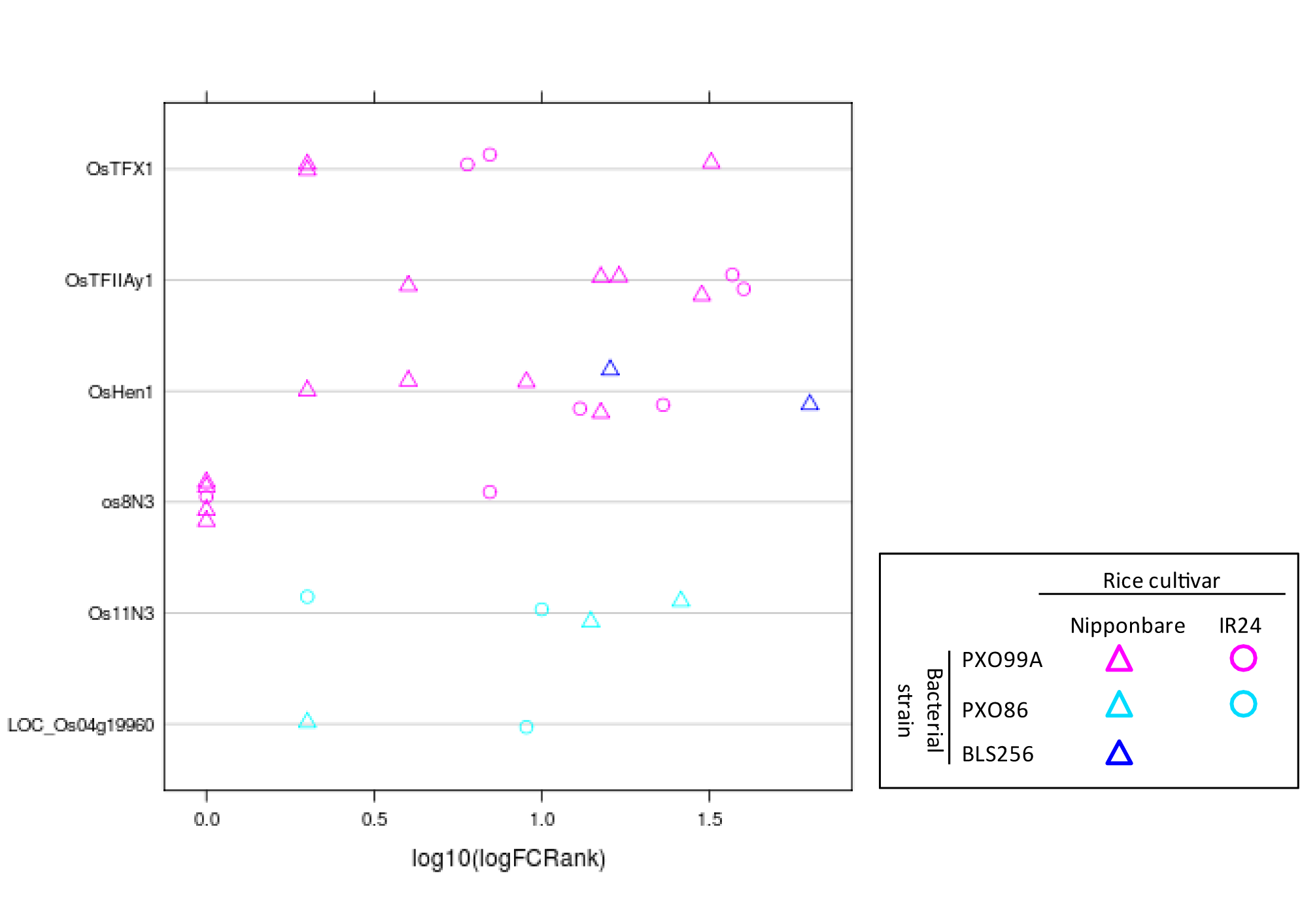

Supplement: Figure S1 — Distribution of the relative expression ranks of control TALE target genes in microarray data. The x-axis values correspond to a decimal log transform of the rank of the log2 fold change (logFCRank) of the Affymetrix probeset(s) corresponding to a control gene in a specific treatment comparison. For all bacterial strain-rice cultivar combinations (see legend), the logFCRank data correspond to a wild type strain versus mock comparison. For PXO99A, a wild type strain versus mutant strain defective for type III secretion comparison was also included. (TIF) [file pone.0068464.s001.tif]

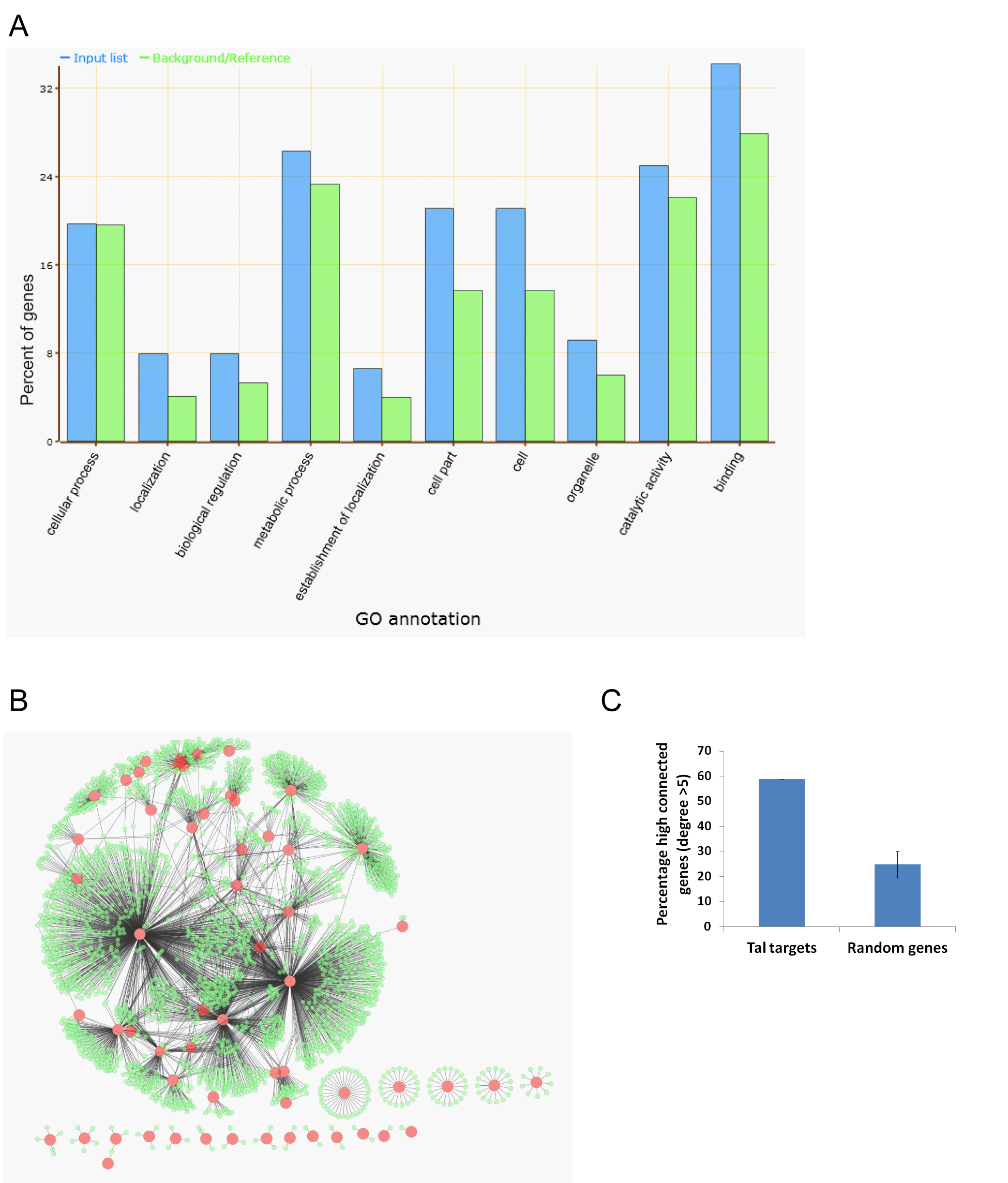

Supplement: Figure S2 — Relevance of candidate TALE target genes in available rice functional prediction data. (A) Singular Enrichment Analyses for GO terms associated with candidate TALE target genes (input list) compared to background annotations in rice performed with AgriGO. No significant differences were found (Fisher exact test, p-value <0.05). (B) Predicted functional network for candidate TALE target genes in the probabilistic functional gene network RiceNet. Candidate TALE target genes are shown in red, non-TALE targets are shown in green (C) Frequency of highly connected genes in RiceNet among candidate TALE targets compared to randomly selected sets of rice genes. (TIF) [file pone.0068464.s002.tif]

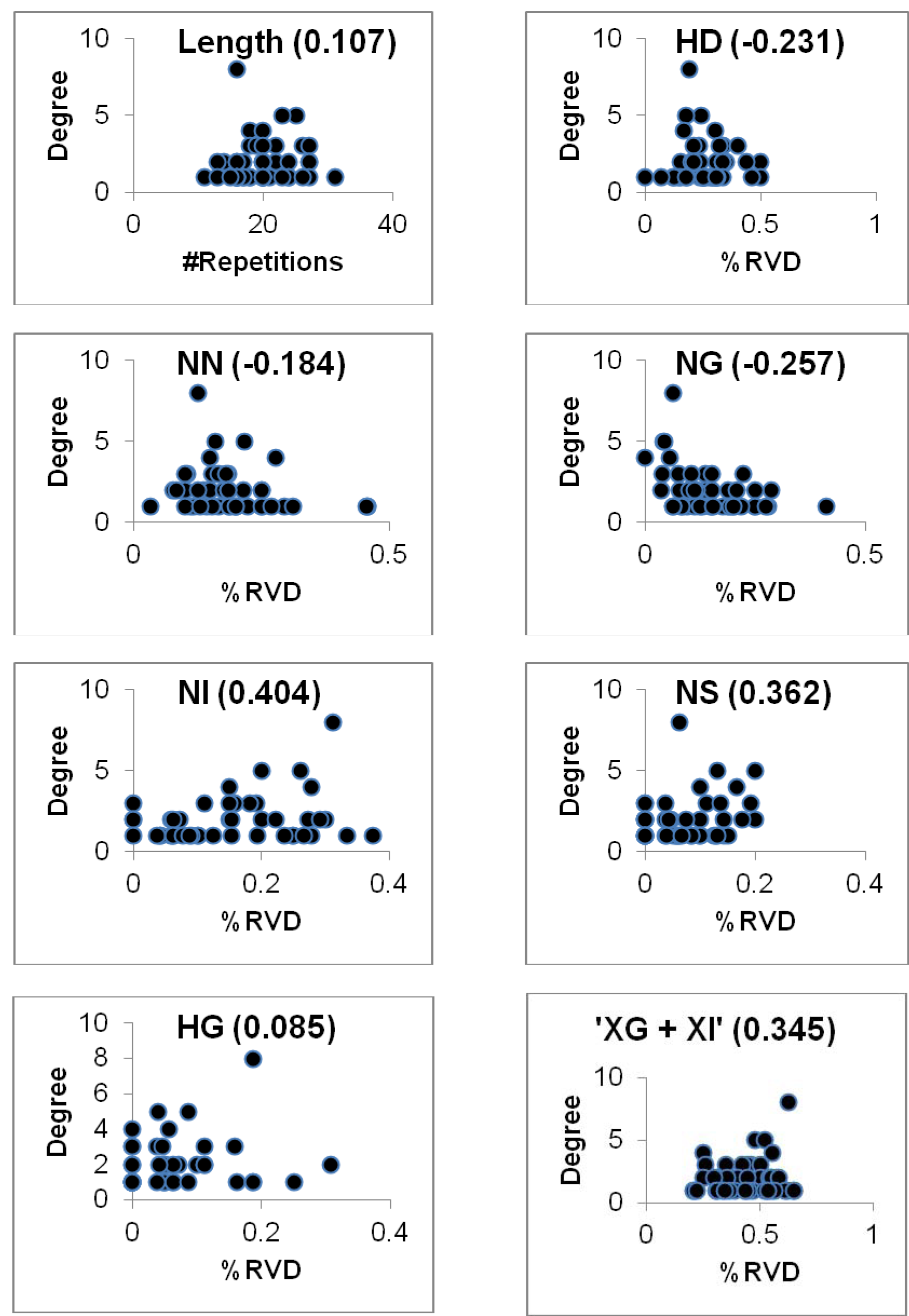

Supplement: Figure S3 — A survey of possible association between in-network TALE degree and TALE RVD sequence features. Dispersion plots showing the relation between TALE degree (total number of targets) and TALE length (in number of repetitions) or TALE common RVDs frequency (percentage) in the predicted TALE-target gene network. Spearman correlation values are shown between parentheses. (TIF) [file pone.0068464.s003.tif]

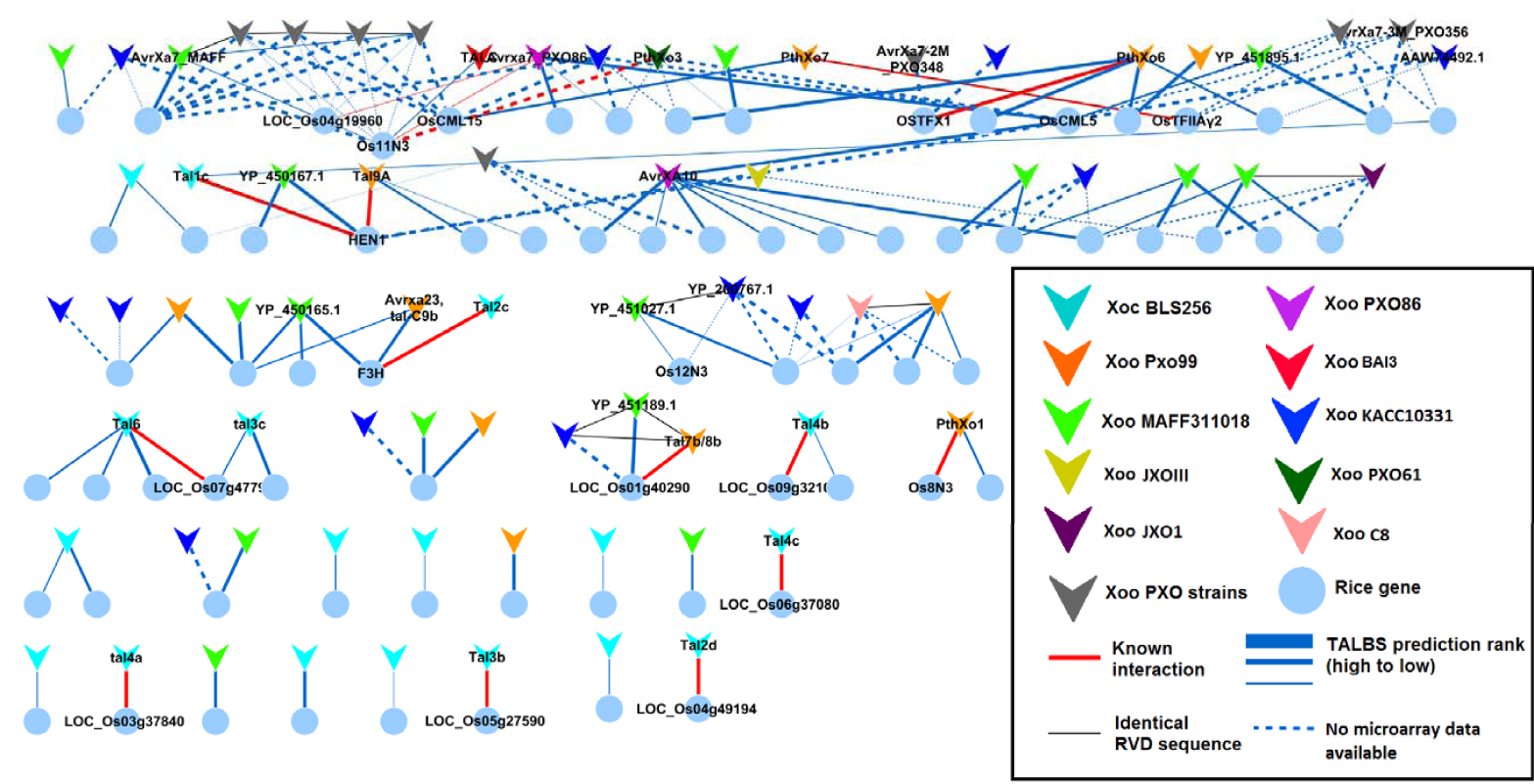

Supplement: Figure S4 — Representation of the “conserved target” network. See the legend of Figure 4A for details. Gray color represent Avrxa7-related TALEs from Xoo strains: KXO85, PXO0314, PXO2648, PXO348, PXO356, PXO357, PXO557 and an in-vitro Avrxa7 mutant. (TIF) [file pone.0068464.s004.tif]
